# Learning Ability and Hippocampal Transcriptome Responses to Early and Later Life Environmental Complexities in Dual-Purpose Chicks

**DOI:** 10.3390/ani12050668

**Published:** 2022-03-07

**Authors:** Chao Yan, Wen Liu, Jinlong Xiao, Hai Xiang, Jikun Wang, Hui Zhang, Jian Liu, Siyu Chen, Xingbo Zhao

**Affiliations:** 1 Shenzhen Branch, Guangdong Laboratory for Lingnan Modern Agriculture, Agricultural Genomics Institute at Shenzhen, Chinese Academy of Agricultural Sciences, Shenzhen 518124, China; yanchao@caas.cn; 2 College of Animal Science and Technology, China Agricultural University, No. 2 Yuanmingyuan West Road, Beijing 100193, China; liuwenwindy159@163.com (W.L.); grdwy123456@163.com (J.X.); emc42021@163.com (H.Z.); 3 Kunpeng Institute of Modern Agriculture at Foshan, Foshan 528200, China; 4 Guizhou Nayong Professor Workstation of China Agricultural University, Bijie 551700, China; vamyluo@126.com (H.X.); liujian8230@163.com (J.L.); 5 Guangdong Provincial Key Laboratory of Animal Molecular Design and Precise Breeding, Key Laboratory of Animal Molecular Design and Precise Breeding of Guangdong Higher Education Institutes, School of Life Science and Engineering, Foshan University, Guangyun Road No. 33, Shishan Town, Nanhai District, Foshan 528231, China; 6 Key Laboratory of Qinghai-Tibetan Plateau Animal Genetic Resource Reservation and Utilization, Ministry of Education, Institute of Qinghai-Tibetan Plateau, Southwest University for Nationalities, Chengdu 610041, China; xdzwjk@163.com

**Keywords:** early and later life, enriched environment, learning, hippocampus, chick

## Abstract

**Simple Summary:**

The hippocampus region of birds is a pivotal area for learning and memory. Early-life conditions can have a long-lasting impact on animals. However, the influence of early-life and later-life environments on animals’ health and welfare has not been well-studied. This study addresses the impact of early-life enrichment and the later environment on the learning ability and hippocampal responses in chicks. We found that the early-life environmental complexity did not prepare better for learning ability in response to life challenges in the future. This study indicates that perches and litter materials, which enriched early-life conditions, were conducive to improved stress responses later in life in terms of neural- and immune-related gene expression and functional pathways. This can be attributed to the “silver spoon” effect. However, the enrichment through litter materials alone in early life does not improve the hippocampal plasticity in later stressed environments. In addition, early-life barren conditions that match later-life conditions have beneficial impacts on neural development, supporting the match and mismatch theory. This study helps us to understand the relationship between early- and later-life environments from the perspective of animal neural- and immune-related development. It also has the potential to guide the treatment of mental problems and personal health in humans based on the interactions between the early-life and later-life environments.

**Abstract:**

In this study, we hypothesized that complex early-life environments enhance the learning ability and the hippocampal plasticity when the individual is faced with future life challenges. Chicks were divided into a barren environment group (BG), a litter materials group (LG), and a perches and litter materials group (PLG) until 31 days of age, and then their learning abilities were tested following further rearing in barren environments for 22 days. In response to the future life challenge, the learning ability showed no differences among the three groups. In the hippocampal KEGG pathways, the LG chicks showed the downregulation of neural-related genes neuronal growth regulator 1 (*NEGR1*) and neurexins (*NRXN1*) in the cell adhesion molecules pathway compared to the BG (*p* < 0.05). Immune-related genes *TLR2* in Malaria and Legionellosis and *IL-18* and *IL18R1* in the TNF signaling pathway were upregulated in the LG compared to in the BG (*p* < 0.05). Compared to the BG, the PLG displayed upregulated TLR2A in Malaria (*p* < 0.05). The PLG showed upregulated neural-related gene, i.e., neuronal acetylcholine receptor subunit alpha-7-like (*CHRNA8*) in the nicotine addiction pathway and secretagogin (*SCGN*) gene expression, as compared to the LG (*p* < 0.05). In conclusion, early-life environmental complexities had limited effects on the learning ability in response to a future life challenge. Early-life perches and litter materials can improve neural- and immune-related gene expression and functional pathways in the hippocampus of chicks.

## 1. Introduction

The hippocampus region in birds is a pivotal area for learning and memory [1,2] and is highly responsive to evolutionary adaptations. Memory and learning ability have a crucial role in the adaptability and fitness of animals. For example, the memory of specific routes and landmarks could be related to the locations of food or dangerous situations [3]. Developing and maintaining learning ability is also associated with neurogenesis and the establishment of neural pathways in the hippocampus [4]. Thus, the development of birds’ hippocampus is crucial for adaptability, fitness, and welfare. Hippocampal volume can be altered through learning processes induced by food-storing activities [5] and migration experience [6] in birds. Environmental complexity influences the hippocampal morphology of adult birds [7,8]. On the contrary, exposure to early-life stress (e.g., a barren system or poor environment) may impact neural plasticity and impair hippocampal plasticity in birds, suggesting that birds in the wild have a complex hippocampus with more neurons than adult aviary birds [4]. These studies indicate that environmental complexity is conducive to hippocampal plasticity in birds. However, how early-life environmental complexity influences hippocampus gene expression and function into later life has not been well-studied in birds. 

Animals retrieve and memorize information from the environment to maximize the cost–benefit ratio, and the environment influences their development and adaptability. Early-life environmental complexity has profound effects on individual abilities, which is partly mediated by developmental plasticity to cope with future life challenges. Furthermore, ontogeny depends not only on the environment during early life, but also on the later-life environment, according to the match–mismatch theory [9,10] and the “silver spoon” hypothesis [10]. The match–mismatch theory suggests that individuals benefit from environments similar to their early environment (i.e., matching conditions), while individuals in a “mismatched environment” suffer poor development [9,10]. The “silver spoon” hypothesis suggests that excellent conditions experienced early in life allow superior development later in life, regardless of the adult environment [10]. 

A wider framework, known as the developmental origins of health and disease (DOHaD), also refers to the conditions of early and later life, life history, and evolutionary concepts. In human beings, postnatal conditions play a vital role in later disease [11]. It points out that “mismatch between the anticipated and the actual mature environment exposes the organism to risk of adverse consequences—the greater the mismatch, the greater the risk” [11]. In addition, the combination of exposure to stress early in life and environmental changes later in life may influence metabolic processes in humans [12]. In rhesus monkeys, a lack of a secure attachment relationship has adverse long-term effects on health, even when they have a normal social environment later in life [13]. In mice, a lack of maternal care during the early-life stage programs the glucocorticoid receptor (GR) gene promoter in the hippocampus, which can be reversed by cross-fostering later in life [14]. Severe maternal deprivation impairs learning ability and neurogenesis in early life, while it can enhance hippocampal functionality in later life [15]. In pigs, early and later life environmental enrichment can affect behavior and growth [16], personality [17], specific antibody responses, and blood leukocyte subpopulations [18]. In birds, rearing in a barren environment causes the long-term impairment of short-term spatial cognition in chickens [19]. Stressful events in early life can alter stress resilience or vulnerability to later-life challenges through the epigenetic regulation of corticotropin-releasing hormone (CRH) [20]. Therefore, in both human beings and non-human beings, the conditions of early life and later life influence organism plasticity and development. However, changes in learning ability and the hippocampal transcriptome profile due to environmental changes between early life and later life have not been well-studied in birds. 

This study, therefore, aimed to investigate the effects of early-life environmental complexity on the learning ability and transcriptome plasticity of the hippocampus of Weining chicks placed in barren conditions later in life. Our study gave new perspectives on how early-life environmental complexity can be optimized to enhance coping and improve animal welfare, even following adaptation due to environmental changes. 

## 2. Materials and Methods

### 2.1. Experimental Design

The trial was performed in an organic farm in Nayong county, Bijie city, Guizhou province, China. One hundred and twenty newly hatched Weining chicks (ratio of male and female = 1:5), a Chinese dual-purpose native breed, were randomly provided by Guizhou Nayong Yuanshengmuye Ltd (Bijie, China). The chicks were equally brooded in three different early-life environmental groups, which were as following: (1) a barren environment group (without enrichment materials) (BG, n = 40); (2) an environment enriched with a litter materials group (wood shavings and sand) (LG, n = 40); and (3) an environment enriched with two perches and a litter materials group (wood shavings and sand) (PLG, n = 40), from post-hatching to 31 days of life. Each group was housed in a unit (2 × 2 × 1.2 m^3^) 0.5 m above the ground with a wire flooring (diameter: 10 mm). Drinkers and feeders were available from hatching onwards. The BG birds had no environmental enrichment. The LG birds had access to substrate litter materials offered on a solid plate (sand (1 × 1 m^2^) and wood shavings (1 × 1 m^2^)), which were replenished daily. Birds in the PLG treatment had two perches (length: 2 m; diameter: 0.05 m), plus the litter material as described for the LG. Each unit was heated by a lamp to guarantee that the temperature exceeded 32 °C from hatching to 16 days of age. The temperature and humidity in the room were recorded every two minutes and measured at least five times, ignoring the highest and lowest, taking the average value. The temperature and humidity were recorded every day during the experiment. The temperatures were 32–35 °C (1–16 days) and 22–28 °C (17–53 days), and the relative humidity was around 60–65%. A comfortable temperature was maintained, so that the chicks were active and did not gather together. Ventilation was through a natural system. The lighting regime was 16 h light followed by 8 h darkness. The chicks were all fed the same diet: a commercial starter feed during the first 30 days, followed by a grower feed (New Hope Group, Chengdu, Sichuan, China) from 31 days to the end of the experiment at 53 days. From 31 days to the end of the experiment, the BG, LG, and PLG were reared in original pens, but in uniform environmental conditions, with two drinkers, two feeders, and no enrichment. 

### 2.2. Data Collection

#### 2.2.1. Learning Test

**Figure 1:**
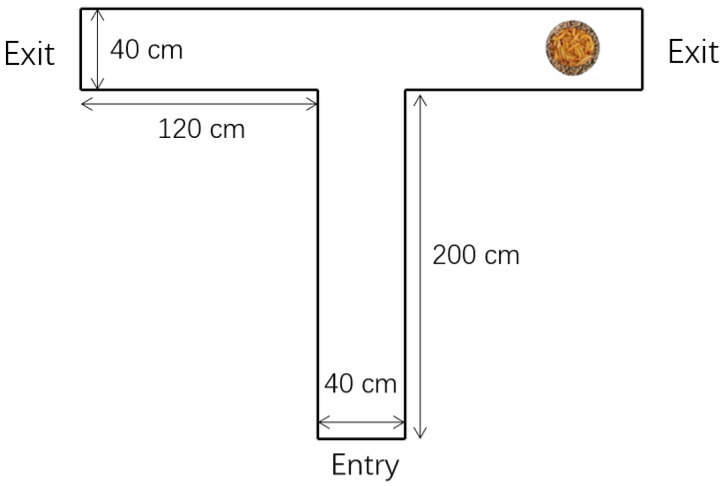
Dimensions of the T-maze. Height of the T-maze: 100 cm.

The learning test was conducted in a T-maze, as previously detailed, with a slight change [21]. Briefly, at 49 and 50 days, 10 female birds in two batches randomly selected from each treatment were marked with a 16 mm foot ring and deprived of food from 18:00 the day before the test and water for 3 h during the testing. The area connected to the start box was 200 cm long, and it was 40 cm wide at the end, where the end turning a 90° to the left or right was located, followed by another 120 cm long arm (Figure 1). The chicks were habituated to the T-maze for 1 h, and there was regular food with live worms available at both ends of the maze during this time. Testing was conducted during the daytime, and food was situated at the far end of one of the arms, out of the chick’s sight. For a test bird, the food was always situated at the same end of the maze, and the direction was balanced in the test. Each test session was recorded by video, until the bird found the food and had fed for 30 s or a maximum of 10 min. The chick was considered to have made a choice when it reached the end of either arm. When a bird chose the objective arm (food existed) first in five out of six consecutive test sessions, it was considered to have solved the task, was recorded as “success” and then was returned to its pen. Birds that failed to habituate and did not reach the food during the initial test session were returned to their home pens, recorded as “failure”, and replaced with a different bird.

#### 2.2.2. Creatine Kinase Concentration

Creatine kinase is a stress sign used to evaluate stress-related physiological responses [22,23]. At 53 days of age, 10 random female birds per treatment were humanely euthanized. Plasma samples were collected and immediately stored in an anticoagulation tube. This was centrifuged at 4000× *g* for 5 min at 4 °C and stored in a 1.5 mL tube at −20 °C. Creatine kinase concentrations (ng/mL) were measured using the commercial assay ELISA kit (ColorfulGene, JYM0024Ch, USA).

#### 2.2.3. Transcriptome Profiles of the Hippocampus

At 53 days of age, 10 randomly selected female birds from each group were humanely euthanized. The right hemisphere of the hippocampus was immediately collected and stored in dry ice, and then at –80 °C until further processing. Total RNA was extracted with the TRIzol reagent (Invitrogen, Life Technologies Corporation, Carlsbad, CA, USA) according to the published protocols. RNA samples were checked by a 2100 Bioanalyzer (Agilent RNA 6000 Nano Kit, Molecular Probes, Inc., Eugene, OR, USA). After checking the quality, cDNA libraries were constructed and prepared using a NEBNext^®^ Ultra™ RNA Library Prep Kit for Illumina^®^ (NEB, Beverly, MA, USA). An Illumina Hiseq platform was used to generate paired-end 150 bp reads. The raw sequences were quality-controlled by removing adapters and low-quality reads. The clean data were aligned to the reference genome Gallus gallus (Ensembl genome browser 97) using the software HISTA v0.1.6-beta [24]. The clean data were aligned to the reference sequence calculated by the Bowtie2 [25]. Then, gene expression level counts were enumerated with RBGM v1.2.12 by the ratio of fragments per kilobase of exon per million fragments mapped (FPKMs) values [26]. Novel genes were discovered and identified based on new transcripts by StringTie and Cufflinks. 

The ratio of FPKM values was used to calculate gene expression levels. The differentially expressed genes (DEGs) were set as a fold change of ≥2 with an adjusted *p*-value of <0.05 (also called the false discovery rate, FDR) [27]. The heat map was created using the package “pheatmap” of software R (v3.1.1), reflecting the hierarchical clustering of samples. Gene ontology (GO) annotation was implemented by using phyper (R package) [28,29]. After obtaining the GO annotation for DEGs, WEGO software was used to perform GO functional classification for DEGs and to understand the distribution of gene functions of the species at the macro level, with a *p*-value of ≤0.05 as the statistical criteria [30]. The enrichment of DEGs in the Kyoto Encyclopedia of Genes and Genomes (KEGG) pathway was analyzed by the phyper (R package), with a *p*-value of ≤0.05 as the statistical criteria. 

### 2.3. Statical Analysis

All data were analyzed using IBM SPSS Statistics 21. The chi-square test was used to analyze learning ability (success or failure) through SAS 9.2 (SAS Inst. Inc., Cary, NC, USA). Creatine kinase values (n = 30; 10 birds/treatment) were checked for the normality and homogeneity of variance and analyzed using the Kruskal–Wallis test. *p*-values of <0.05 were regarded as statistically significant. Data are presented as means ± SE (Standard Error). 

## 3. Results

### 3.1. Learning Ability

**Figure 2:**
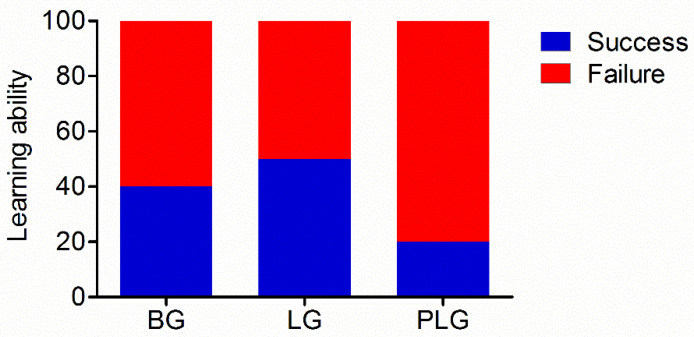
Learning ability in the future barren life challenge. BG, barren environment group; LG, environment enriched with a litter materials group; PLG, environment enriched with perches and a litter materials group.

Learning ability was not statistically different among the BG (mean rank: 13.50), LG (mean rank: 15.00), and PLG (mean rank: 18.00) (χ^2^ = 1.94, *p* = 0.38). The percentages of success were 40%, 50%, and 20% in the BG, LG, and PLG, respectively (Figure 2).

### 3.2. Plasma Hormone Concentrations

**Figure 3:**
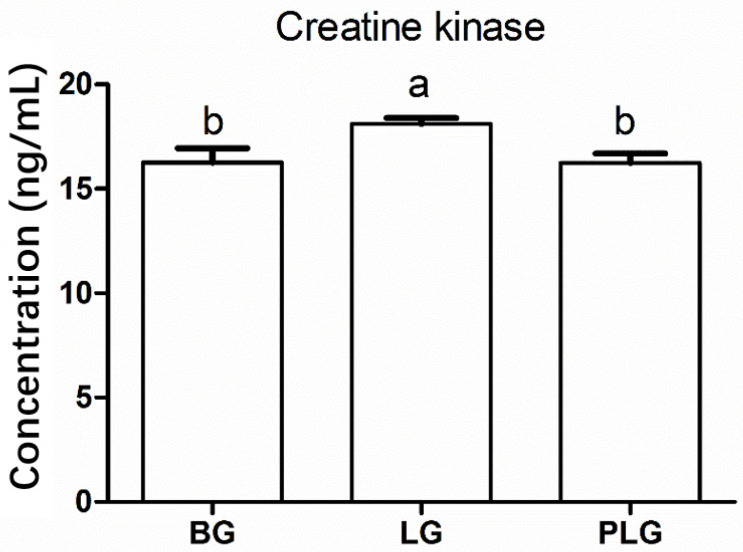
The plasma creatine kinase levels (in ng/mL) of chicks in the barren life challenge, for the three treatment groups: BG, barren environment group; LG, environment enriched with a litter materials group; PLG, environment enriched with perches and a litter materials group. Bars with different letters indicate that the means were statistically different (*p* < 0.05).

At 53 days of age, creatine kinase concentrations (means ± SE, ng/mL) in the BG (16.26 ± 0.68), PLG (16.24 ± 0.45), and LG birds (18.11 ± 0.27) were significantly different (*p* = 0.034). The LG birds (18.11 ± 0.27) were higher as compared to the BG (*p* = 0.023) and the PLG (*p* = 0.026), whereas the BG and the PLG did not differ from each other (Figure 3). 

### 3.3. Hippocampal Transcriptome

#### 3.3.1. DEGs

**Figure 4:**
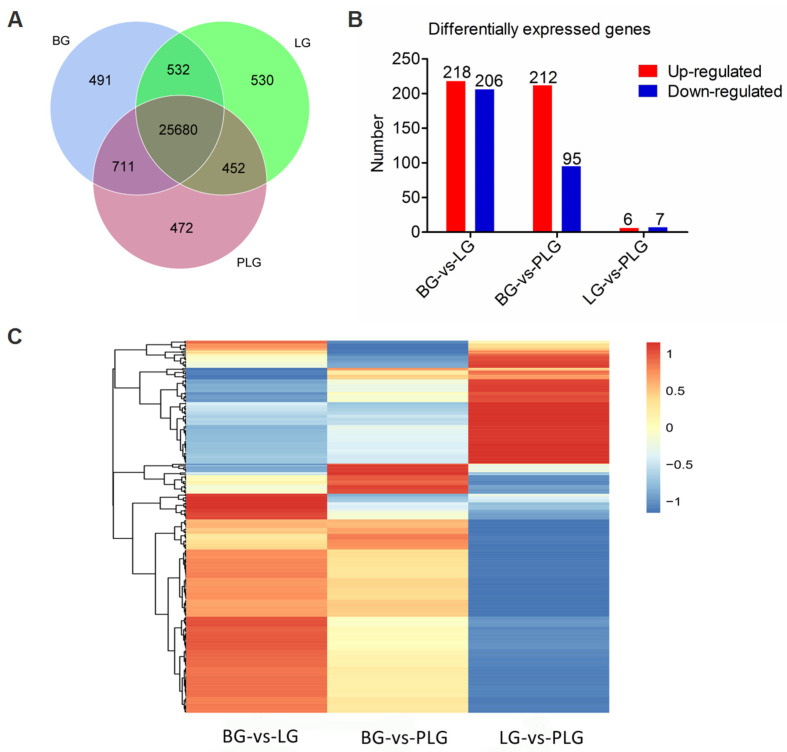
Hippocampal transcriptome. (**A**) Venn diagram for the BG, the LG, and the PLG. (**B**) Differently expressed genes (DEGs) among treatment groups (fold change ≥ 2, adjusted *p*-value < 0.05). (**C**) Heat map of DEGs. BG, barren environment group; LG, environment enriched with a litter materials group; PLG, environment enriched with perches and a litter materials group.

The 30 hippocampal samples generated, on average, 6,1768,598 total clean reads among the BG, LG, and PLG. Of these reads, 85.12% were mapped onto the reference genome, with 83.63% being paired. The gene expressions among the three groups are shown in Figure 4A. The Venn diagram (Figure 4A) displayed a co-expression of 26,212 genes and specifically expressed 1006 genes in the BG and 1202 genes in the LG. The genes showed a co-expression of 26,391 genes between the PLG and LG and specifically expressed 924 genes in the PLG and 1023 genes in the LG. The genes showed 26,156 co-expressions between the BG and the PLG and specifically expressed 1062 in the BG and 1159 in the PLG. 

DEGs are shown in Figure 4B (fold change ≥ 2, adjusted *p*-value < 0.05). We obtained 424 DEGs, including 218 genes that were upregulated and 206 genes that were downregulated in the LG as compared to in the BG. We obtained 307 DEGs, including 212 genes that were upregulated and 95 genes that were downregulated in the PLG as compared to in the BG. We obtained 13 DEGs, including six genes that were upregulated and seven genes that were downregulated in the PLG as compared to in the LG. The DEGs are shown in the heat map (Figure 4C). 

#### 3.3.2. GO Terms 

**Figure 5:**
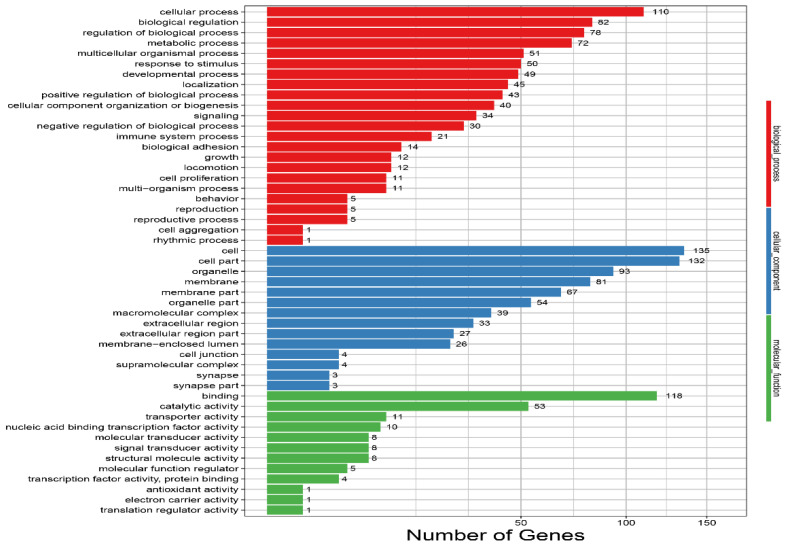
Enriched gene ontology (GO) terms for the BG and the LG. The vertical axis indicates GO terms, and the horizontal axis represents the number of DEGs. The numbers of enriched genes in each GO term are shown in the biological process (red color), cellular component (blue color), and molecular function (green color) areas. BG, barren environment group; LG, environment enriched with a litter materials group.

The GO annotation analysis of the DEGs was divided into three classifications: biological process, cellular component, and molecular function. The DEGs were more enriched in the biological process area, including the metabolic process (72 genes), response to stimuli (50 genes), reproductive process (five genes), growth (12 genes), developmental process (49 genes), localization (45 genes), response to stimulus (50 genes), immune system process (21 genes), locomotion (12 genes), and behavior (five genes, including LMX1A (ENSGALT00000005411.6), HTR2B (ENSGALT00000012451.6), neuronal growth regulator 1 (NEGR1; ENSGALT00000018520.3 and ENSGALT00000093001.1), and ZIC1 (ENSGALT00000056152.2)) in the LG compared to in the BG. Three DEGs were enriched in the synapse part (cellular component area), and three DEGs were enriched in the synapse, in the LG compared to in the BG (Figure 5; Table S1 in Supplementary Materials). 

**Figure 6:**
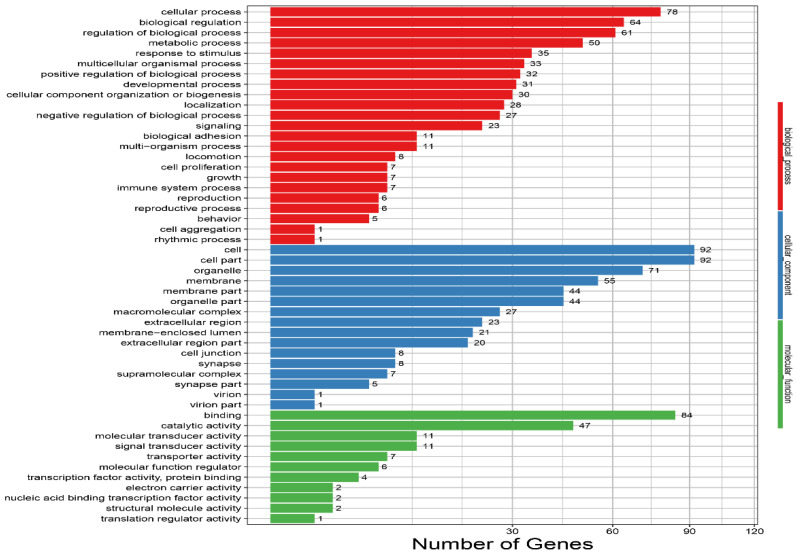
Enriched GO terms for the BG and the PLG. The vertical axis indicates GO terms, and the horizontal axis represents the number of DEGs. The numbers of enriched genes in each GO term are shown in the biological process (red color), cellular component (blue color), and molecular function (green color) areas. BG, barren environment group; PLG, environment enriched with perches and a litter materials group.

The DEGs were more enriched in the biological process area, including the growth (seven genes), reproductive process (six genes), immune system process (seven genes), reproduction (six genes), localization (28 genes), response to stimulus (35 genes), locomotion (eight genes), behavior (five genes), and developmental process (31 genes), in the PLG as compared to in the BG. Eight DEGs were enriched in the synapse (cellular component area), and five genes were enriched in the synapse part (cellular component area) in the PLG as compared to in the BG (Figure 6; Table S2 in Supplementary Materials). 

**Figure 7:**
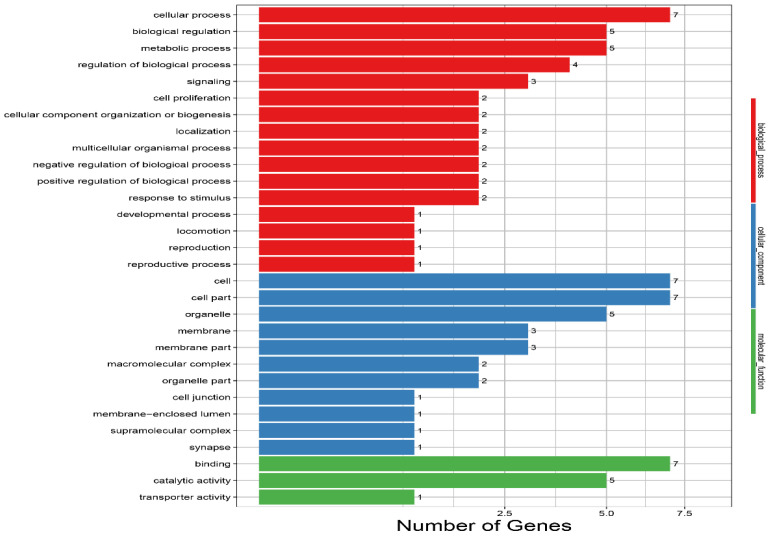
Enriched GO terms for the LG and the PLG. The vertical axis indicates GO terms, and the horizontal axis represents the number of DEGs. The numbers of enriched genes in each GO term are shown in the biological process (red color) area, cellular component (blue color) area, and molecular function (green color) area. LG, environment enriched with a litter materials group; PLG, environment enriched with perches and a litter materials group.

One DEG was enriched in the immune system process, and one DEG was enriched in the response to stimulus attributed to the biological process, in the PLG as compared to in the LG. One DEG was enriched in the synapse (cellular component area) in the PLG as compared to in the LG (Figure 7; Table S3 in Supplementary Materials).

#### 3.3.3. KEGG Pathway

Compared to in the BG, *NEGR1* and neurexins (*NRXN1*) in the cell adhesion molecules were downregulated in the LG (*p* < 0.05; Table 1). *TLR2* in Malaria and Legionellosis and *IL-18* and *IL18R1* in the TNF signaling pathway were upregulated in the LG compared to in the BG (*p* < 0.05; Table 1). 

Compared to in the BG, neuronal acetylcholine receptor subunit alpha-7-like (*CHRNA8*), associated with nicotine addiction, was upregulated, while glucokinase (*GCK*), associated with amino sugar and nucleotide sugar metabolism, was downregulated in the PLG (*p* < 0.05; Table 2). Compared to the BG, PLG chicks displayed an upregulated *TLR2A* in Malaria (*p* < 0.05; Table 2). 

The upregulation in the LG as compared to in the PLG was observed for secretagogin (*SCGN*), which is associated with endocrine and other factor-regulated calcium reabsorption, and 3-hydroxy-3-methylglutaryl coenzyme A synthase 1 (*HMGCS1*), which is associated with synthesis and degradation of ketone bodies, butanoate metabolism, terpenoid backbone biosynthesis, valine, leucine, and isoleucine degradation (*p* < 0.05; Table 3).

## 4. Discussion

For early-life environmental complexity, substrate and perches allow the expression of natural behavior in chickens and have a positive impact on chicks’ early-life behavioral development and welfare [31,32,33,34,35]. In the later barren condition challenge, no significant difference was observed among the three groups in learning ability or mRNA expression of genes in the hippocampus related to learning ability, including N-methyl-D-aspartic acid (*NMDA*) [36,37] and brain-derived neurotrophic factor (*BDNF*) [38,39]. Enriched environments generally exert a positive effect on learning performance in laboratory animals and farm animals [40,41]. Due to environmental changes from early life to later life, our results are inconsistent with previous studies that demonstrated early-life barren environments adversely impair [19] and enriched environments improve learning ability in birds [32]. Our findings may be in line with a study that found that short-term (seven days) enrichment in housing conditions had a positive effect on learning performance but did not have a long-lasting effect after a memory test [42]. Our findings are inconsistent with earlier findings that early- and later-life conditions can modulate learning ability in rats [12]. Based on our results, learning ability in a future barren conditions challenge may not be improved through early-life environmental complexity. 

In general, plasma creatine kinase, a stress indicator hormone, increases, when an individual is exposed to external stress (e.g., heat stress) [43]. The creatine kinase concentrations in the BG and the PLG were lower than in the LG, suggesting higher stress in chicks reared in a substrate enriched environment, which was in line with a previous study involving corticosterone [44]. The later-life mismatch did not explain why the levels in PLG were similar to those in the BG. Our results are inconsistent with previous studies that demonstrated an early-life barren environment adversely impairs the stress response. The inconsistency with previous findings may potentially be due to the change in the environments from early life to later life. A possible explanation for this is that the beneficial effects of early-life enrichment are transient and overruled by later-life challenges. Nevertheless, our results do support the match–mismatch theory [9,10], as barren-reared chicks performed equally well as enriched reared chicks in a barren environment. In this case, enriched chicks experienced a mismatch, but a matched environment was provided for the barren-reared chicks. The results showed support for the “silver-spoon” theory [10], in that perches with litter materials did have a benefit in later life. 

In the later-life barren condition, differences did exist in the hippocampal gene expression of chicks, especially between the barren-reared chicks and both enriched groups. The heat map revealed that the exposure to various enrichment materials did influence the gene expressions of the hippocampus in later life. In rats, these long-term effects are, at least in part, mediated by epigenetic alterations in the hippocampus [14]. In our study, early-life environmental complexity also influenced the transcriptome profile in a later-life challenge. When it comes to KEGG pathways, environmental complexity can influence neural plasticity. Regarding the neural development, *NEGR1* [45] and *NRXN1* [46] in the cell adhesion molecules pathway are mainly associated with neural growth and synaptic plasticity and were found to be upregulated in the BG as compared to in the LG. Meanwhile, the DEGs were enriched in the synapse part of the GO terms in the LG as compared to in the BG. The previous literature has indicated that an enriched environment improves neural development and brain plasticity [36,47], while early-life adversity or stress may increase the risk of neurodevelopmental disorders and induce a reduction in hippocampal plasticity, with studies including humans [48] and birds [4]. In our study, the neural plasticity in the BG and the LG was inconsistent with those previous findings. Our findings again agreed with the match–mismatch effect between the BG and the LG. In addition, the DEGs enriched in the localization, locomotion, and behavior of the GO terms may respond to the memory or learning ability differences between the LG and the BG, although the learning abilities were not different, and previous studies have proven that early-life environments influence learning ability [19,32]. Our results indicated that early-life environmental effects influencing learning ability were not long-lasting until later-life period. The DEGs enriched in the reproductive process, developmental process, and growth relating to fitness are influenced by environmental complexity [10]. When it comes to the response to the stimulus and the immune system process of GO terms, combined with KEGG pathways, DEGs were enriched in the pathways in cancer, Legionellosis, Malaria, and the TNF signaling pathway in the LG compared to in the BG. *TLR2*, *IL-18*, and *IL18R1* were upregulated in the LG compared to in the BG. *TLR2*, as one of the important components of the innate immune response, has a pivotal role in the early recognition of pathogens, as well as in the initiation of a robust and specific adaptive immune response in chickens [49]. Previously, early-life adversity was found to be detrimental to the immune phenotype [50], which is similar to the results for the BG. Additionally, early-life environmental complexity is associated with the immune development and the stimulation response in broilers and layers [51]. Thus, the results related to the immune system illustrated that early-life litter materials may improve the immune development, consistent with the “silver spoon” theory. 

*CHRNA8* (also known as *CHRNA7L*) is involved in neuronal survival and synaptic plasticity [52] and was upregulated in the PLG as compared to in the BG. *GCK*, which was increased in the PLG as compared to in the BG, plays an important role in maintaining blood glucose homeostasis by increasing insulin release and promoting glucose utilization to provide energy [53]. A more enriched environment can lead to more complex neural brain structures [7]. At the same time, the DEGs were enriched in the synapse part and synapse of the cellular component at GO terms. The development and maintenance of knowledge through learning may be costly in terms of energy required for neurogenesis and the establishment of neural pathways [54]. Our results suggest that the neural development of the PLG was better than that of the BG, despite the later barren environment being relatively worse for the PLG. Similarly, in GO terms, when the PLG was compared to the BG, the DEGs were enriched in the growth, reproductive process, reproduction, and developmental process relating to fitness, localization, locomotion, and behavior associated with memory and learning ability areas, with the LG sharing the same trend with the BG. However, learning ability was not significantly different, which may be attributed to the early-life limited effect for memory in our study. Additionally, DEGs were enriched in the immune system process and response to stimulus areas in the GO terms, and *TLR2A* was enriched in Malaria in the KEGG pathways. From this, we can conclude that early-life enriched environments are conducive to the immune system [55], and further evidence for the positive effect of an early-life enriched environment was found based on the PLG. 

Meanwhile, *SCGN*, as a novel neuroendocrine marker [56], was upregulated in the PLG as compared to in the LG in the endocrine and other factor-regulated calcium reabsorption pathways. *HMGCS1*, which acts as a control enzyme in cholesterol synthesis, was upregulated in the PLG when compared to the LG in pathways including the synthesis and degradation of ketone bodies, butanoate metabolism, and terpenoid backbone biosynthesis [57]. Cholesterol is abundant in the central nervous system and is involved in dendrite outgrowth, the hyperplasia of the stellate cell, and the development and remodeling of nerves [58,59]. DEGs were enriched in the synapse of the cellular component at the GO term. Additionally, the downregulated gene ENSGALT00000003855.5 was enriched in the immune system process, and the response to stimulus attributed to the biological process, in the PLG as compared to in the LG. Thus, the PLG did not result in vulnerability but promoted the resilience capacity of nerves which equips organisms to respond more effectively when facing the challenges of barren conditions later in life [60]. Hence, the PLG showed positive adaptations, which means encountering perches and litter materials in early life may prepare the birds for dealing with the later-life environmental context, which is a crucial factor for animal development [61]. Overall, the hippocampal gene expression suggests a “silver spoon” effect, whereby perches and litter materials in early life improve the neural plasticity in the hippocampus, leading to better coping abilities in the later-life barren condition challenge. 

This study initially explored how early- and later-life environmental complexities regulated the learning ability and the hippocampal plasticity. However, the limitations of this paper include the lack of a balanced treatment for early good–late good and early poor–late good groups. In addition, the mechanisms of early- and later-life environmental complexities were not identified, especially in terms of epigenetic regulation. Epigenetics is closely linked to host phenotype responses to the effects of the environment [62]. For example, an associated study indicated that the regulation of the GR gene promoter methylation [14], as well as CRH histone modification and DNA methylation [20], can modulate plasticity and metabolic homeostasis. Thus, future studies should pay more attention to mechanisms by which environmental factors influence phenotypic plasticity and adaptive programming. 

## 5. Conclusions

Early-life environments influencing learning ability are not a long-lasting effect in a future life challenge. Based on the results of hippocampal gene expression, an environment with perches and litter materials in early life offered the condition for the more optimal development of neural-related (CHRNA8) and immune-related (TLR2A) gene expressions, thereby possibly supporting the “silver-spoon” theory. Chicks given early-life litter materials showed improvements only in immune-related gene expressions (TLR2, IL-18, and IL18R1) and functional pathways in a barren environment challenge, which may be attributed to the mismatching effect. Overall, this study contributes to an increased understanding of the role of the early-life environmental complexity in the learning ability and hippocampal plasticity of chicks that are later kept in barren conditions. 

## Figures and Tables

**Table 1:** DEGs enriched in Kyoto Encyclopedia of Genes and Genomes (KEGG) pathways in the BG and the LG.

BG—LG ^1^	DEGs	
	Upregulated	Downregulated
Fatty acid biosynthesis	*ACSL5*	
Primary bile acid biosynthesis	*CH25H*, *BAA*	
TNF signaling pathway	*VCAM1*, *CASP18*, *MAP3K5, IL-18*, *IL18R1*, and *BGLE*	*TRAF5*
Cell adhesion molecules	*VCAM1*, *TROJAN*, *ITGBL1, PTPRK*, *BGLECTIN*, *MHC-I*, *SIGLEC1*, and *ICOS*	neuronal growth regulator 1 (*NEGR1*) and neurexins (*NRXN1*)
Apoptosis	*CRCBL*, *CTSS*, *CASP18*, and *MAP3K5*	*RAPGEF4*, *PTPN13*, and *KNDC1*
Regulation of actin cytoskeleton	*ITGBL1*, *AKAIN1*, *SASH3, MYL9, LPXN*, and *APC*	*RASGEF1A*, *ARPC1A, ABI2*, *FGF12*, *ARHGEF4*, *DAZAP2*, and *KNDC1*
Malaria	*TLR2*, *VCAM1*, *CR1L*, *BGLP*, and *BGLE*	
Legionellosis	*TLR2*, *CR1L*, and *CASP18*	
Pathways in cancer	*ITGBL1*, *CASP18*, *AGTR1, SASH3*, *SPI1*, *ADCY8*, *GLI2, CDKN2A*, *GMSRAL*, *APC*, *CSF2RA*, *LAMA3/5*, and *NIPA1*	*TRAF5*, *FGF12*, *RASGEF1A*, *RUNX1*, and *KNDC1*

^1^ indicates that the latter group was compared to the former group. BG, barren environment group; LG, environment enriched with a litter materials group. Only genes that significantly differed at *p* < 0.05 are shown.

**Table 2:** DEGs enriched in KEGG pathways in the BG and the PLG.

BG—PLG ^1^	DEGs	
	Upregulated	Downregulated
Proteasome		*PSMB7*
Amino sugar and nucleotide sugar metabolism		*UXS1*, glucokinase (*GCK*), *GPI*, and *GFPT1*
VEGF signaling pathway		*AKT2*, *MAPKAPK3*, and *TGFB1I1*
Regulation of lipolysis in adipocytes	*MGLL*	*AKT2*
Malaria	*TLR2A*	
Nicotine addiction	Neuronal acetylcholine receptor subunit alpha-7-like (*CHRNA8*)	*EHD2* and *GRIA4*

^1^ indicates that the latter group was compared to the former group. BG, barren environment group; PLG, environment enriched with perches and a litter materials group. Only genes that significantly differed at *p* < 0.05 are shown.

**Table 3:** DEGs enriched in KEGG pathways in the LG and the PLG.

LG—PLG ^1^	DEGs	
	Upregulated	Downregulated
Synthesis and degradation of ketone bodies	*HMGCS1*	
Butanoate metabolism	*HMGCS1*	
Terpenoid backbone biosynthesis	*HMGCS1*	
Homologous recombination	*NBN*	
Endocrine and other factor-regulated calcium reabsorption	Secretagogin (*SCGN*)	
Aminoacyl-tRNA biosynthesis		*KARS*
Valine, leucine and isoleucine degradation	*HMGCS1*	

^1^ indicates that the latter group was compared to the former group. LG, environment enriched with a litter materials group; PLG, environment enriched with perches and a litter materials group. Only genes that significantly differed at *p* < 0.05 are shown.

## Data Availability

The transcriptome’s raw sequencing data generated in this study are openly available in the National Center for Biotechnology Information (PRJNA663230).

## Supplementary Materials

The following supporting information can be downloaded at: https://www.mdpi.com/article/10.3390/ani12050668/s1. Table S1. GO terms in the BG vs. the LG; Table S2. GO terms in the BG vs. the PLG. Table S3. GO terms in the LG vs. the PLG.
